# Activation induced cytidine deaminase: An old friend with new faces

**DOI:** 10.3389/fimmu.2022.965312

**Published:** 2022-10-27

**Authors:** Elif Çakan, Gurcan Gunaydin

**Affiliations:** ^1^ Hacettepe University School of Medicine, Sihhiye, Ankara, Turkey; ^2^ Department of Basic Oncology, Hacettepe University Cancer Institute, Sihhiye, Ankara, Turkey

**Keywords:** AID, SHM, CSR, central B cell tolerance, epigenetic heterogeneity, lymphomagenesis

## Abstract

Activation induced cytidine deaminase (AID) protein is a member of APOBEC family. AID converts cytidine to uracil, which is a key step for somatic hypermutation (SHM) and class switch recombination (CSR). AID also plays critical roles in B cell precursor stages, removing polyreactive B cells from immune repertoire. Since the main function of AID is inducing point mutations, dysregulation can lead to increased mutation load, translocations, disturbed genomic integrity, and lymphomagenesis. As such, expression of AID as well as its function is controlled strictly at various molecular steps. Other members of the APOBEC family also play crucial roles during carcinogenesis. Considering all these functions, AID represents a bridge, linking chronic inflammation to carcinogenesis and immune deficiencies to autoimmune manifestations.

## Introduction

The APOBEC (apolipoprotein B mRNA editing enzyme, catalytic polypeptide-like) protein family represents a group of evolutionarily conserved cytidine deaminases, which can deaminate cytosine residues in DNA and RNA, resulting in somatic mutations, DNA breaks, RNA modifications or DNA demethylation (1–3). Indeed, these proteins demonstrate diverse and vital functions in both human health and diseases. Although these enzymes are essential for physiological immune (*e.g.* protecting humans from viral infections) and non-immune processes, they might function to promote tumor evolution and drive poor disease outcomes in various malignancies (4). As such, APOBEC mutations have been demonstrated in several cancers. On the other hand, they may also be implicated in autoimmune diseases, diabetes, and triple nucleotide repeat diseases. Different members of the APOBEC family utilize similar deamination activities to achieve various biological effects. While deamination mediated by APOBEC-3 has the potential to inhibit the replication of retroviruses (*e.g.* human immunodeficiency virus), deamination by activation-induced cytidine deaminase (AID), is crucial for generating high affinity antibodies (5).

Among eleven APOBEC proteins that have been described in humans, AID is the evolutionary founding member (6). AID/APOBECs family phylogenetically originates from a branch of the zinc-dependent deaminase family at the beginning of the vertebrate radiation (7, 8). AID, which is encoded by the *AICDA* gene in humans, has also been implicated in active DNA demethylation *via* deaminating 5-methylcytosine, which can then be replaced with cytosine by base excision repair (9). In addition to its pivotal roles in physiological processes, defects in normal AID functioning are associated with several clinical conditions such as Hyper-IgM syndrome type 2 as well as lymphomagenesis (10–12).

In this Review, we provide a general overview of AID functions in physiology as well as during the course of several diseases, underlining its genomic and epigenomic impact. We will thoroughly discuss the role of AID in antibody diversification, emphasizing the regulation of AID activity. We will discuss the novel functions of AID in detail, such as elimination of polyreactive B cells from repertoire and the role of AID in lymphomagenesis as well as non-lymphoid oncogenesis. AID can also drive epigenetic heterogeneity, affecting the prognosis of and treatment response in diffuse large B-cell lymphoma (DLBCL) and possibly in other malignancies. Last but not least, we propose AID to be at the crossroads between immune deficiencies and autoimmunity as well as inflammation and carcinogenesis. Such a pivotal role should be kept in mind especially during the evaluation of common variable immune deficiency (CVID) patients in clinical practice, since they might also have autoimmune conditions, or *vice versa*. For instance, patients with systemic sclerosis and/or systemic lupus erythematosus (SLE) were demonstrated to have deficient B cell activation response after Toll-like receptor (TLR)-9 stimulation (13, 14).

**Figure 1:**
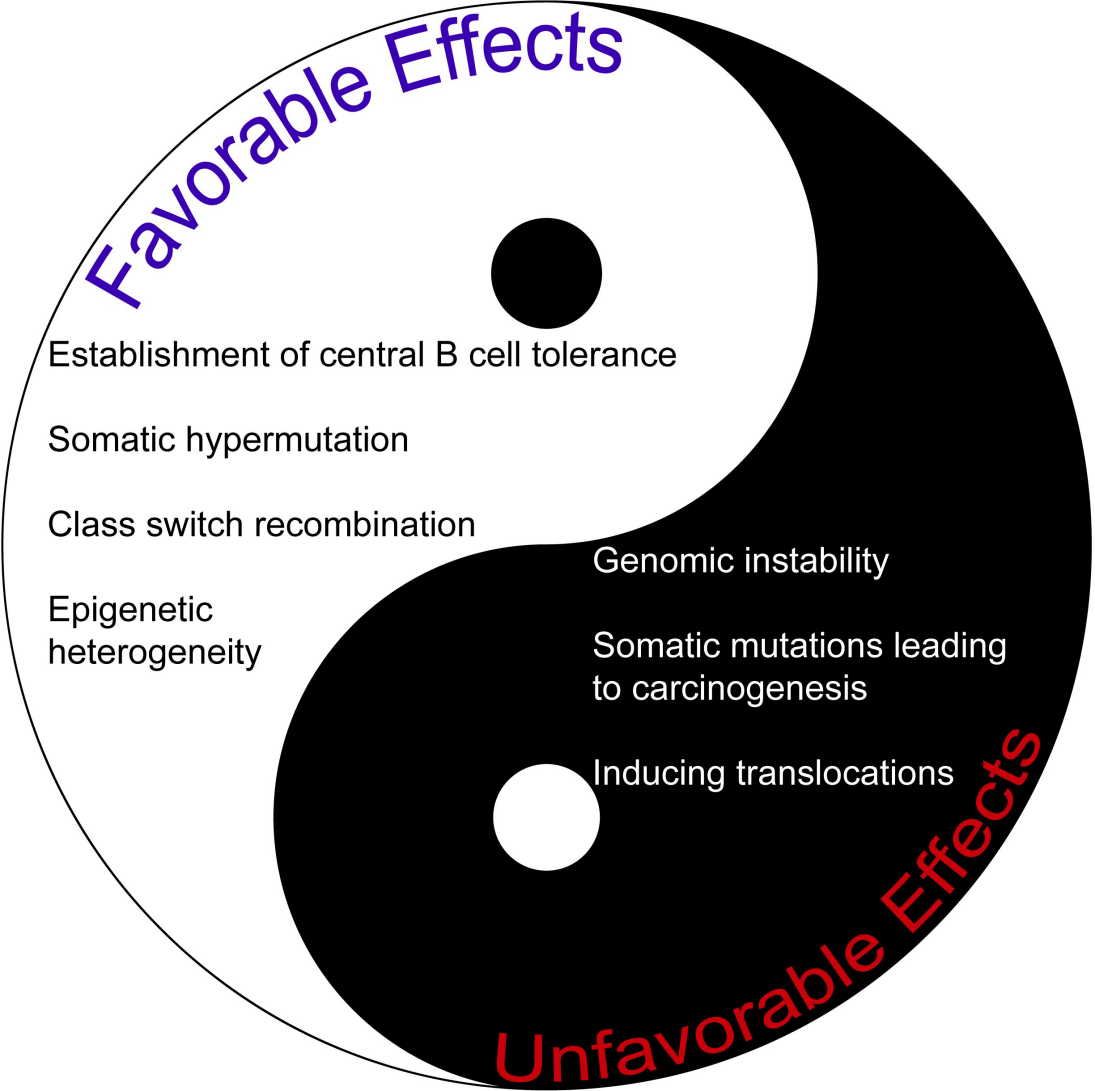
The comparison of favorable and unfavorable effects of AID. AID may be regarded as a *double-edged sword* given its plethora of activities.

Given its vast number of intriguing roles, AID may indeed serve as a *double-edged* sword (**Figure 1**). Thus, we believe novel and pioneering studies that aim to understand the full potential of AID in terms of clinical translation are undoubtedly exciting. A better understanding of novel concepts about AID function in both physiology and pathology will be very useful for pursuing and improving effective therapeutic strategies in the future, especially during the precision medicine era.

## The old friend of B cells: AID is a key molecule to augment antibody diversity

Producing extremely diverse antibodies (estimated to be in excess of 10^9), which is significantly more than the coding capacity of the inherited genome is essential for effective humoral immune responses (15). The main mechanisms to increase the variability of immunoglobulins are mediated through V(D)J gene recombination and somatic hypermutation (16–19). During the early stages of B cell development, RAG recombinase binds to the specific recombination signal sequences (RSS) in the variable (V), diversity (D) and joining (J) genes. Then, it cleaves the DNA, followed by recombination and DNA repair, mostly by classical non-homologous end joining (NHEJ) repair pathway, generating functional and extraordinarily diverse array of antigen receptors (20–26). Following V(D)J recombination, the antibody diversity is further enhanced by either somatic hypermutation (SHM) (human and mice) or gene conversion (chicken, rabbits, cattle and pigs) and class switch recombination (CSR) of heavy chains, which produces B cells secreting antibodies with different effector functions (15, 27). The cycling between proliferation in the dark zone and selection in the light zone of germinal centers represents the best way to generate high affinity antibodies (28, 29). Lately, with the help of improved visualization and genetic tools, our understanding of the germinal center structure and dynamics has been improved greatly, but there are still lots of exciting questions to be answered (30–38).

In 1999, Muramutsu et al. showed the structure of AID gene, which is homologous to APOBEC-1, bearing cytosine deaminase activity. They also demonstrated that AID was expressed specifically in the germinal centers (39). After this seminal discovery, several supportive findings from mice and humans demonstrated that AID is essential for both CSR and SHM mechanisms. For instance, AID ^-/-^ C57BL/6 mice displayed complete absence of class switching before or after immunization with T-dependent antigens and also abrogated hypermutation of the specific VH gene (40). In humans, mutations of huAID are responsible for the Hyper-IgM syndrome type 2 (HIGM2), which is characterized by lack of or very low levels of serum IgG and IgA, absence of IgV somatic mutations, lymphadenopathy and tonsillar hypertrophy, in contrast to healthy donors (41).

The first phase of SHM is maintained by the AID-induced mutations at G:C bases, whereas the second phase depends on the error-prone base excision repair (BER) and mismatch repair (MMR) mechanisms (more at A:T bases) (42). In contrast to general functions of BER and MMR enzymes providing genomic stability, they become error-prone in the germinal center centroblast B cells by low-fidelity translesional DNA polymerases such as Pol η, ι and REV1, which have more permissive binding sites. This represents an important regulatory mechanism for the AID targeting. Several other candidate DNA polymerases (pols θ, λ, μ, ν) need to be further investigated for their functions as translesion synthesis polymerases (43). Recently, an interesting study demonstrated that FAM72A protein regulated physiological uracil-DNA glycosylase (UNG) 2 level and; therefore, balancing the error-prone and error-free DNA repairs.

The initiating step of CSR is the deamination of dC nucleotides by AID within S regions, which contain numerous AID hot spots (44, 45). It should be noted that 10 amino acids of AID are required specifically for CSR, which might be due to the interaction with essential mediators (46, 47). Later, the dU residues are removed by UNG and abasic sites are repaired by apurinic/apyrimidinic endonuclease, producing single stranded breaks (SSBs) (44, 48). If the SSBs are in close proximity on opposite DNA strands, they can spontaneously form a double stranded break (DSB). If not, MMR could help distal SSBs to form a DSB (49). DSBs in the donor and acceptor S regions are combined by the NHEJ process as well as Ku70, Ku80 and two-protein ligase complex XRcc4-ligase IV are crucial for this mechanism (50).

## Regulation of AID

**Figure 2:**
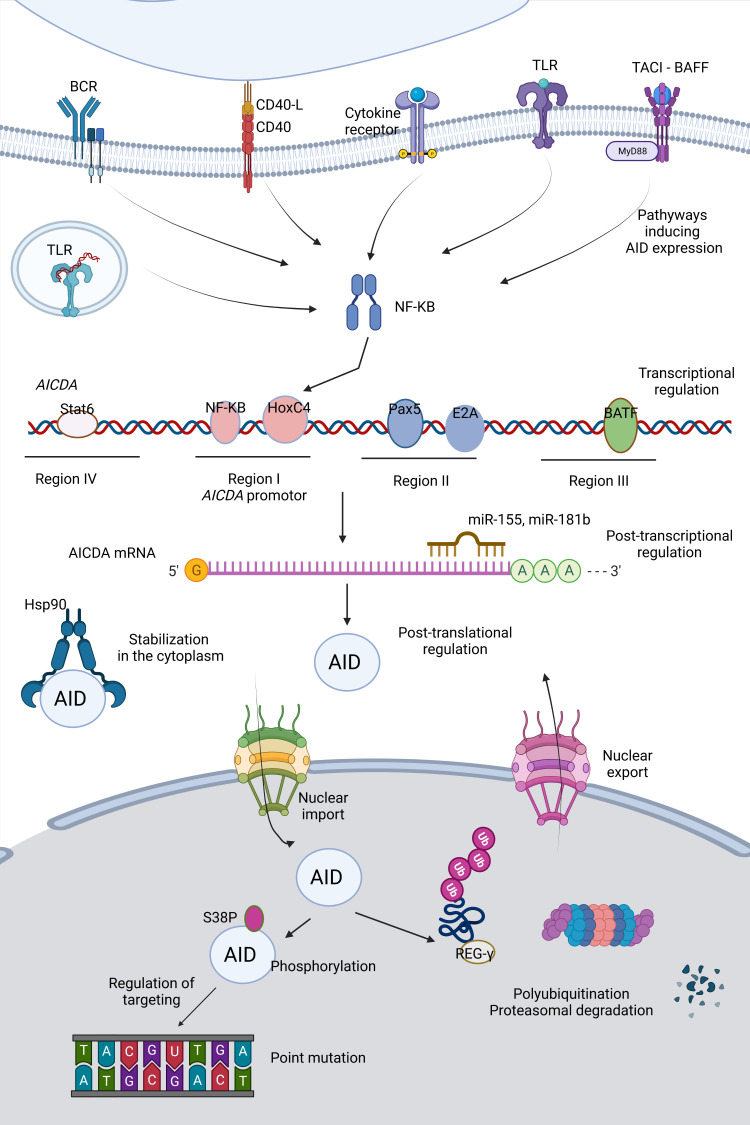
Regulation of AID. Activity as well as expression of AID is tightly regulated at various levels (*e.g.* transcription, post-transcription and post-translation).

AID is a critical molecule for an effective immune response, as impaired AID function can result in immunodeficiency or autoimmunity. However, it also represents a potential mutator; thus, off-target activity of AID is a significant threat to the genetic integrity and can lead to increased mutation burden, translocations, and oncogenesis. Therefore, the regulation of AID should be maintained very strictly at the transcriptional, post-transcriptional, post-translational levels and in terms of targeting and enzymatic function (**Figure 2**).

In early transitional B cells, co-activation of TLRs and B cell receptor (BCR); in activated mature B cells, CD154:CD40 engagement are primary inducers for AID expression through activation of the NF- κB pathways (51–53). NF-κB p52 subunit (non-canonical) is recruited to the promoter of *AICDA* gene, which encodes AID, and p65 subunit (canonical) is recruited to an upstream enhancer element (54). Activated NF-κB heterodimers translocate to the nucleus and synergize with HoxC4 and SP1, SP3 transcription factors (55). Cytokines secreted by T cells during B:T cell interactions such as IL-4 and TGF-β are the secondary inducing stimuli directing isotype selection (56). Paired box protein 5 (Pax5), E2A proteins, Smad3/4 and AP1 family transcription factor BATF are important transcription factors mediating AID expression (54, 57). Two binding sites for Myb, one binding site for the E2F and a 350 bp CT-rich sequence are negative regulators of AID transcription (54). 3’ untranslated region (UTR) of *AICDA* has binding sites for miR-155 and miR-181b, which control the AID expression levels and protect B cells from increased mutation load (58, 59).

The distribution of AID between the cytoplasm and nucleus determines the AID activity and genome stability. Active nuclear export provides restricted AID levels in the nucleus. Additionally, AID becomes a constant target to proteasomes by polyubiquitination in the nucleus, decreasing its stability, whereas heat shock protein 90 kD (Hsp90) prevents it from degradation in the cytoplasm (60–63). AID phosphorylation at Ser38 and Tyr184 is an important step for the function of AID specific to B cells, which is enriched in the chromatin-associated nuclear fraction (64–66). Phosphorylation of AID can be linked to its localization in the nucleus (67). The nuclear/cytoplasmic distribution of AID is also controlled by a variety of AID co-factors such as eEF1A, GANP, CTNNBL1, Hsp90, PKA, REGγ (68). β-catenin like protein 1 (CTNNBL1) is known to interact with AID and mediate localized cytidine deamination; however, it is not clear whether it is functional for nuclear shuffling or bringing AID to its targets by close association with transcribed genes (69). Recently, a patient with CVID associated with autoimmune cytopenias (AIC) with progressive severe hypogammaglobulinemia has been described to carry a single M466V amino acid replacement in the CTNNBL1 protein. Decreased AID binding to the CTNNBL1 466V variant was shown to result in impaired nuclear translocation of AID and reduced SHM and CSR functions (70).

AID tends to mutate DNA/RNA hybrids composed of S region sequences (71).The S regions undergoing recombination in IgH locus have high frequency of 5’-AGCT-3’ repeats, preferred substrate of AID and also targets for 14-3-3 adaptor proteins mediating CSR. Open chromatin state of these regions recruiting CSR factors and AID-binding proteins such as Spt5 and Spt6, PTPB2, and RNA exosomes provide selective targeting by the CSR machinery (68). Single-stranded DNA-binding protein RPA stabilizes the interactions of phosphorylated AID and target DNA (72).

Transcription itself is an important regulator for the targeting of AID activity, increasing the accessibility of DNA due to chromatin remodeling and generating the dsDNA. AID activity is inclined to supercoiled dsDNA rather than linear dsDNA, but it could still mutate both, although with lower efficiency for the latter (73). The protein structure of AID is crucial for the regulation of its activity. Indeed, improved techniques helped us to understand the crystal structure (74). dC should access and fit to the catalytic pocket. In addition, the catalytic pocket should be in an energetically favorable conformation for the deamination, explaining the low catalytic rate of AID. Larijani et al. revealed that only 0.7%-8.0% of AID-DNA interactions resulted in dC deamination (75, 76).

### Epigenetic regulation of AID

The expression of AID can be modulated *via* DNA methylation, histone modifications, and miRNAs in B cells (77). Promoter DNA hypermethylation is able to mediate suppression of *AICDA* expression in B lymphocytes (78). Histone H3 acetylation was reported to occur in the *AICDA* gene at low levels comparable to the overall H3 acetylation in the genome and neighboring genes in naive B cells (79). In contrast, DNA of the *AICDA* gene is demethylated and the locus becomes enriched in H3K4me3 and H3K9ac/K14ac after activation of B cells (79). Such epigenetic alterations may assume pivotal roles in terms of activating *AICDA* transcription. Moreover, remethylation of the *AICDA* DNA might lead to down-regulation of *AICDA* transcription after SHM and/or CSR (80). Additionally, methylation of cytidines can provide a protection of DNA from the mutator capacity of AID. However, methylation may be unable to provide a global safeguard against AID-mediated activity, since methylation might not protect cytidines that neighbor CpG motifs (81).

Furthermore, various miRNAs may demonstrate important roles in regulating AID expression. For instance, miR-155 may target several genes, including Myd88, Pu.1 as well as *AICDA*, and affect B cell differentiation (82–84). miR-16 might decrease AID and Blimp expression in B cells (85). In addition to miR-155 and miR-16, miR-181b and miR-361 can also regulate AID expression by binding to target sites in the 3′ UTR of *AICDA* mRNA, resulting in decreased *AICDA* mRNA and AID protein levels (83, 84, 86–88). Such miRNAs may function to reduce AID expression in B cells after SHM and/or CSR or in naïve B cells. It should be noted that miR-155 seems to be one of the most important miRNAs that reduce AID expression. In fact, pre-miRNA-155 and mature miR-155 sequences were reported to be conserved across various organisms such as birds, amphibians as well as mammals (83, 89).

## New faces: Emerging functions and the untold story of AID

AID was classically thought to be solely expressed in the germinal center, contributing to increased antibody diversity. However, it was found that AID is also expressed in early developmental stages of the B cells. The expression is further stimulated if the B cell carries an autoreactive BCR recognizing self-antigens, inducing numerous point mutations, which drive these autoreactive B cells to apoptosis and removal from the repertoire. On the other hand, off-target activity of AID can lead to gene translocations and increased mutational load playing an important role for lymphomagenesis and carcinogenesis. Another interesting potential function of AID is DNA demethylation *via* deamination of 5hmCs, although it requires further studies to unravel such effects. These dynamic aspects of AID need to be further investigated and bear potentials for better understanding the pathophysiology of several diseases as well as developing alternative therapeutic strategies.

### AID expression in early transitional B cells leads to elimination of polyreactive B cells from the repertoire

**Figure 3:**
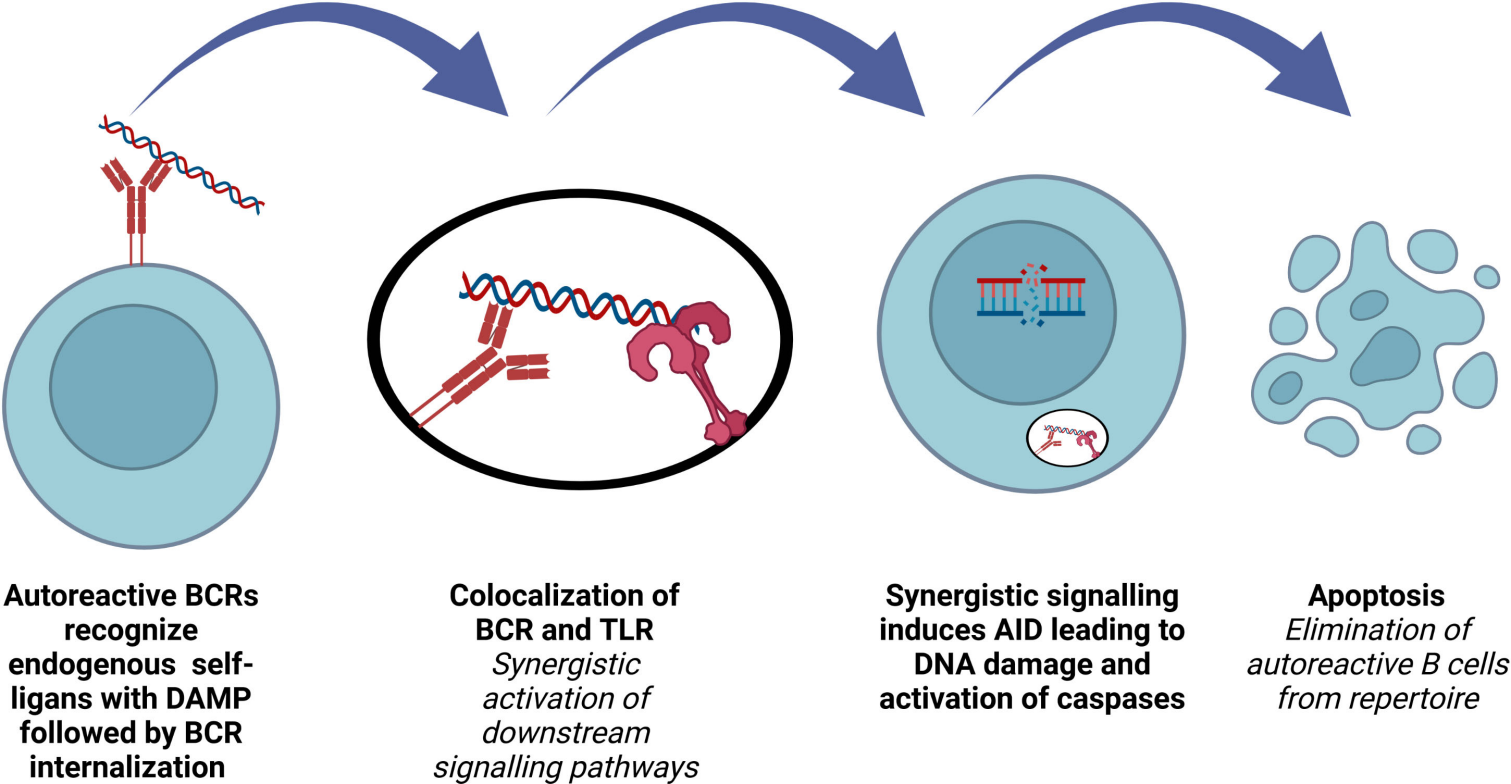
Role of AID in terms of B cell repertoire. Elimination of polyreactive B cells from immune repertoire involves AID expression in early transitional B lymphocytes.

Increasing the BCR diversity to encompass all microbial pathogens by VDJ recombination, SHM and CSR is crucial for B cell development and function. A functional BCR production, which does not recognize self-antigens, is the major control point of this process. Inactivation of autoreactive B cells occurs first in the bone marrow, at central tolerance checkpoint. Then, peripheral tolerance by means of receptor editing, anergy and deletion take place after B cells travel to the periphery (90). The first checkpoint is regulated by B cell intrinsic factors such as BCR, TLRs, whereas the second checkpoint is more dependent on extrinsic factors such as regulatory T cells and microbiota derived factors (90–93). Understanding the regulation of these two checkpoints is crucial in order to enlighten the pathophysiology underlying autoimmune and autoinflammatory diseases as well as numerous important biological processes (**Figure 3**).

The discovery of AID expression in several B cell precursors was a surprising observation, since AID had been known to be expressed only in mature B cells as a key molecule for SHM and CSR. Such an expression was transient and restricted to a minor population of early immature B cells, co-expressing recombination-activating gene 2 (Rag2). AID^+^ immature B cells lacked MCL-1 and expressed active caspase-3 (94–96). It was also found that *AICDA* ^-/-^ immature B cells are significantly more resistant to tolerization and BCR-induced apoptosis, explaining the impaired central B cell tolerance and elevated dsDNA auto-ab levels in *AICDA* ^-/-^ mice (97). Antibodies cloned from new emigrant B cells from AID-deficient patients and from NSG mice transplanted with hematopoietic stem cells carrying GFP-tagged AID shRNA showed increased frequency of polyreactive clones, supporting the key role of AID for the regulation of the central B cell tolerance (94, 95, 98).

The stimulation of rheumatoid factor (RF) positive B cells with IgG2a a/j monoclonal antibodies depends on both the antibody and the nature of the autoantigen. B cells from RF^+^ MYD88-deficient mice are not responsive to anti-nucleosome monoclonal antibodies, showing that synergistic engagement of BCR and a MYD88-dependent receptor is necessary (99). Impaired regulation of central B cell tolerance checkpoint in patients who are deficient for IRAK-4, MYD88 and UNC-93B proves that endocytic TLR signaling plays a role for the silencing of autoreactive B cells (100). Co-localization of internalized BCR and TLR in an autophagosome-like compartment and synergistic activation of downstream signaling induce high levels of AID expression in immature/T1 B cells, purging the autoreactive clones. Endosomal acidification and MYD88 are required for this process (101). TACI regulates TLR7, TLR9 and BCR functions in B cells and both TACI-deficient and TACI-mutated patients have defective removal of polyreactive B cells and antinuclear clones (102).

TLR9 function is impaired and the regulation of the early B cell tolerance checkpoint is defective in SLE and systemic sclerosis (SSc) patients (14, 103, 104). TLR9 is required for the production of autoantibodies to DNA-containing antigens (105, 106). TLR9-deficient mice have increased immune activation, accelerated lupus nephritis and mortality, whereas TLR7-deficient lupus-prone mice are protected from autoimmune disease; and renal disease was ameliorated as well (106). The opposing roles of TLR7 and TLR9 in the establishment of the central B cell tolerance and how this regulation is impaired in autoimmune syndromes such as SLE and SSc need to be addressed for a better understanding of the mechanisms of these diseases and to develop alternative therapeutic strategies.

### AID/APOBEC family potentially plays a role in DNA demethylation

Reprogramming the methylation patterns is important for genomic plasticity and transdifferentiation, which occurs at least in germ cells and preimplantation embryos, but various cells can also be reprogrammed back to totipotency (107, 108). AID and APOBEC1 are thought to play roles in DNA demethylation *via* their 5meC deaminase activity. The fact that AID and APOBEC1 were found to be co-organized and co-expressed with a cluster of pluripotency genes in oocytes and primordial germ cells (PGC), might be related to their potential roles for DNA demethylation (9). Fused mouse embryonic stem cells and human fibroblasts, which are interspecies heterokaryons, initiate reprogramming towards pluripotency, expressing both human OCT4 and NANOG. A persistent knockdown of AID reduced the demethylation of OCT4 and NANOG promoters and inhibited the induction of these genes. Moreover, this effect was reverted by the overexpression of human AID. ChIp analysis revealed that AID binds to hypermethylated but not active and demethylated OCT4 and NANOG promoters, supporting the involvement of AID in DNA demethylation (109).

TET1 protein catalyzes the oxidation of 5-methylcytosines (5mCs) to 5-hydroxymethylcytosines (5hmCs), which is the target for the active demethylation mechanism in mammalian cells in both CpG and non-CpG contexts (110, 111). However, none of the known mammalian DNA glycosylases possesses 5hmC glycosylase activity. Human AID/APOBEC deaminases can remove the 5hmCs, but they do not show any effects on 5mCs demethylation. Similarities between 5hmCs demethylation and AID/APOBEC mediated deamination such as high processivity, sequence selectivity, transcription dependency and DNA strand preference support the TET1/APOBEC-mediated oxidation-deamination mechanism of active DNA demethylation in mammals (111). The active role of TET for DNA demethylation suggests that even stable epigenetic states are continuously regulated and further investigations are needed to decipher how the fate of cell plasticity is controlled either by reprogramming or by driving stably differentiated cell types (112).

Overexpression of AID/APOBEC enzymes (5-meC deaminase) or MBD4 (G:T mismatch-specific thymine glycosylase) alone does not have any effect on DNA methylation status; however, co-expression of them provides DNA demethylation *via* a G:T intermediate, which could be detected by PCR. Physical coupling of AID/APOBEC with MBD4, which has a methylated-DNA binding domain, rapidly removes the mutagenic thymine by BER (113). Another study revealed that thymine DNA glycosylase (TDG) has glycosylase activity on 5-hydromethyluracil. It interacts with AID and GADD45a, regulates the AID levels, and also protects from aberrant hypermethylation (114). It should be noted that MBD4 has a methylated-DNA binding domain in addition to a glycosylase domain (115). GADD45 proteins, which are activated by DNA damage, are important to promote DNA demethylation and to upregulate specific AID/APOBEC proteins (113, 116). GADD45 proteins do not demonstrate clear enzymatic activity, but they act as essential adaptors for AID/MBD4 complex formation (117).

Comparing the DNA methylation levels in wild type or AID^-/-^ C57BL/6J mice revealed that AID deficiency affected the genome-wide methylation levels only in PGCs, not in the fetus, placenta or sperm (118). Further studies are necessary to reveal the possible contributors of restriction of DNA demethylation activity of AID in PGCs or in specific genetic locations. Since their involvement is restricted to PGCs, the significance of DNA demethylation *via* AID/APOBEC proteins for epigenetic regulation is still not clear.

Teater et al. showed that DLBCLs with high *AICDA* expression have increased cytosine methylation heterogeneity, but not somatic mutation burden (119). Such a finding seems very interesting to open up new avenues in terms of the involvement of AID in DNA demethylation. SHM might as well be mediated by different AID regions or complexes. Additionally, it highlights the possible role of DNA demethylation function of AID/APOBEC family as a driver of epigenetic heterogeneity for other types of tumors, which may affect the prognosis and response to treatment.

## AID in lymphomagenesis

In addition to its various crucial physiological functions, AID may entail a carcinogenic effect due to off-target genomic mutagenesis. DNA editing resembles a *double-edged* sword. It is important for genome defense, but uncontrolled activity can increase the mutation load in somatic cells, leading to cancer (120, 121). Most somatic mutations in cancer cells are passenger mutations, but some are driver mutations in oncogenic genes (122). Kataegis, foci of localized substitution hypermutation, is characterized by C>T and/or C>G mutations and is often associated with genomic rearrangements (123). Alexandrov et al. defined kataegis as six or more consecutive mutations with average intermutation distance of ≤1 kb (123, 124). A comprehensive study by Bergstrom et al. investigating 30 different types of cancer revealed that 76.1% of all kataegic events exhibited mutational patterns associated with AID and APOBEC3 (125).

B cell lymphoma cell lines have higher genomic uracil levels compared to other human transformed cell lines, which correlated with higher AID expression. AID-YFP overexpression in mouse lymphoma cells (CH12F3) lead to increased genomic uracil levels, supporting this correlation. Sequencing the kataegis regions of B cell lymphoma and CLL for C to T mutation revealed a target sequence overlapping with the known AID hotspot motif (WRCY) (126). Additionally, AID can play a role in the translocation mechanisms, especially for the ones that cannot be explained by VDJ gene recombination (127, 128). AID has the ability to bind to G-loops formed within the transcribed c-Myc gene and c-Myc promoter region is essential for recruiting AID to induce DSB formation leading to c-Myc/IgH translocations (129, 130).

Double-hit lymphoma (DHL) is known to carry c-Myc and bcl-2 or bcl-6 gene translocations and has much lower rate of complete response to traditional R-CHOP therapy (around 20%) (131). A study with DHL and DLBCL patient samples showed that AID expression and CSR are significantly higher in patients with DHL. Supporting these findings, stimulation of OCI-Ly18 DHL cells with LPS and IL-4 to induce CSR enhanced AID expression and c-Myc translocation. As a result, AID can promote CSR and lead to c-Myc translocation and is a potential target for treatment in DHL patients (132). Moreover, screening the multiple myeloma samples revealed two different mutation signatures, one of which is an APOBEC signature. The myeloma samples carrying APOBEC signature are highly enriched for the maf translocations, t(14:16) and t(14:20), higher mutational load and lower progression free survival and overall survival (133).

### Diffuse large B cell lymphoma

DLBCL, representing approximately 30-40% of all cases, is the leading subtype of non-Hodgkin lymphoma. It has heterogeneous genetic and clinical features, is characterized by a diffuse proliferation of large and mature B cells and occurs in single or multiple, nodal or extra-nodal sites. The most common classification is based on cell-of-origin, *i.e.* as germinal center B-cell like (GCB) and activated B-cell like (ABC) subtypes, leaving 10-15% cases unclassified (134–136). Patients with GCB-DLBCL have better overall and event-free survivals than ABC-DLBCL group, with R-CHOP therapy (137). Therefore, aiming to decipher the reasons underlying why ABC-DLBCL group is more resistant to therapy and developing new strategies would be useful to improve the prognosis and survival.

Due to genetic heterogeneity of DLBCL, the classification of the disease that correlated with clinical presentation has been challenging. Two seminal studies that utilized new genetic tools, suggested new classifications for DLBCL. Whole exome sequencing of 304 DLBCL patients was performed to detect mutations, SCNAs, SVs and the approach identified five groups of patients. C5 DLBCLs had the highest cAID signature and SHM in contrast to C1, which had low or aberrant cAID activity and lower SHM, suggesting an extrafollicular origin. Spontaneous deamination at CpG sites was the predominant mutational signature. The other two mutational signatures were AID-driven, *i.e.* canonical AID (cAID) and AID2, reflecting different repair mechanisms after AID-induced deamination (138). A similar study classified DLBCLs into MCD (co-occurrence of MYD88L266P and CD79B mutations), N1(NOTCH1 mutations), BN2 (BCL6 fusions and NOTCH2 mutations) and EZB (EZH2 mutations and BCL2 translocations) subgroups, the latter two groups having favorable survival (139). As a result, analyzing larger numbers of DLBCL samples and using new genetic techniques may lead to a better classification that displays a stronger correlation with clinical outcomes. Additionally, investigating the genetic background of therapy resistant groups will open up new avenues to novel alternative targeted therapeutic approaches.

AID expression in ABC DLBCL subgroup was found to be higher than GCB DLBCL group through the analysis of cDNA microarrays from patient samples and by RT-PCR analyses of cell lines. However, there is no correlation between AID levels and number of additional intraclonal IgV_H_ somatic mutations (140, 141). The reasons underlying how increased AID expression did not lead to increased SHM in ABC DLBCL, whereas it is positively correlated in GCB DLBCLs should be further investigated.

Mutation screening of DLBCL samples showed mutations that are unique to individual tumor DNAs in PIM1, MYC, RoH/TTF, and PAX5 gene regions. Lack of hypermutation in these four genes during normal B-cell development confirms that it represents a tumor-associated event and is not common to all germinal-center derived tumors (12). Studies with more advanced genomic tools demonstrated that 25% of genes expressed in germinal centers were mutated compared to AID^-/-^ background value, but less than the immunoglobulin heavy chain JH4 intronic region. Such findings suggest that high fidelity DNA repair mechanisms prevent the accumulation of somatic hypermutations, besides targeted AID activity (142, 143).

Another study demonstrated that AID played a key role in generating cytosine methylation heterogeneity in DLBCLs and was associated with increased tumor fitness, greater inter- and intra-tumor heterogeneity as well as poor outcome. However, it was not found to be associated with increased mutational burden. In a cohort of 63 primary DLBCL patients, *AICDA*-high DLBCLs were more likely to be classified as the ABC subtype, supporting the previous findings, and possibly explaining the reason for poorer outcome compared to GCB-DLBCLs (119). Non-mutational epigenetic reprogramming is one of the four additional features in terms of the hallmarks of cancer (144). Owing to its inter- and intra-tumor heterogeneity inducing capacity, AID/APOBEC family has the potential to affect the pathogenesis and clinical presentation, *e.g.* prognosis and response to treatment, of lymphomas as well as other malignancies.

## AID in non-lymphoid oncogenesis

Bladder, cervical, lung squamous cell carcinoma, lung adenocarcinoma, head and neck cancers and breast cancers bear the most elevated APOBEC3B expressions among 19 different cancer types compared to wild type tissue samples. Positive correlations with APOBEC3B expression levels and the mutations at CG base pairs, the overall mutation load and number of kataegis events are suggestive for the APOBEC3B dependent mutagenesis (145). Similar observations were reported when 2,680 exomes from 14 cancer types were analyzed, mostly from TCGA, additionally revealing APOBEC signature mutations with cancer driver genes (146). APOBEC3B expression was higher in esophageal squamous cell carcinoma samples and was associated with C-to-T transitions of PIK3CA and general DNA hypomethylation, but not with clinical outcome (147).

The similarity of yeast and breast cancer kataegis as well as increased APOBEC3B and APOBEC3A expressions in breast cancer samples are highly suggestive for the role of APOBEC3B and/or APOBEC3A in the breast cancer hypermutation (148). APOBEC3B levels are higher in breast cancer cell lines and patient samples. In addition, APOBEC3B is positively correlated with C-to-T mutation load, overall base substitution mutation load, and TP53 inactivation (149, 150).

Among head and neck squamous cell cancers (HNSCC), HPV^+^ group has higher APOBEC activity as well as A3A and A3B expressions, whereas HPV^-^ group is associated with the smoking-associated mutational signature. Higher A3G or A3H expression is correlated with better overall survival in HPV^+^ HNSSC group. In contrast, A3F gene expression is correlated with better prognosis in the HPV^-^ HNSSC group. Varying effects of APOBECs in HPV^+^ and HPV^-^ HNSCC groups remain to be explained (151–156).

p53 regulates the expression of APOBEC3B through a p21-dependent mechanism and by the E2F4/RB-containing DREAM (dimerization partner, RB-like, E2F and MuvB) repressive complex. p53 suppresses the APOBEC3B expression, leading to decreased mutational capacity of cancer cells. The subversion of this effect by HPV E6 and E7 genes mediates repression of p53, increases the A3B expression and activity, likely playing a key role for the tumor development and tumor evolution (157).

These findings show that AID and other members of the AID/APOBEC family may contribute to non-lymphoid oncogenesis, in addition to their implications in lymphomagenesis. It should be investigated whether APOBEC members may induce cytosine methylation heterogeneity, affect clinical presentation and response to therapy in non-lymphoid cancers.

## AID connects immune deficiencies to autoimmunity as well as inflammation to carcinogenesis

Hyper-IgM syndrome is characterized by increased serum IgM levels and absence of IgG, IgA and IgE, and clinically presents with early onset of severe recurrent sinopulmonary infections, cholangitis associated with persistent cryptosporidium infection, with or without neutropenia. The X-linked form is caused by CD40L or IKK-gamma (NEMO) gene mutations, whereas the autosomal deficient form is due to the *AICDA*, *CD40* and *UNG* gene mutations (41, 158). AID mutations in these patients lead to absence of CSR, SHM and lymph node hyperplasia with intense apoptosis (41).

Since the co-localization of BCR and TLRs is crucial for the induction of AID in autoreactive B cells as well as establishment of central B cell tolerance, patients carrying IRAK-4, MYD88, UNC-93B and TACI mutations have increased numbers of polyreactive B cells in addition to their immunodeficiency syndromes. On the other hand, systemic sclerosis and SLE patients have defective B cell stimulation with TLR9 ligands, in addition to their autoimmune manifestations. Therefore, it seems reasonable to suggest that patients carrying abnormalities in TLR, BCR, and/or AID pathways should be investigated both for B cell stimulation functions and for reactivity to self-antigens, since those pathways are involved both in elimination of autoreactive B cells in early developmental stages and also in B cell stimulation in later stages. It is yet not fully clear which TLRs are critical for the establishment of central B cell tolerance, though.

A study investigating the CVID patients with autoimmunity demonstrated that patients carrying heterozygous *AICDA* gene mutations had impaired peripheral B cell tolerance. However, UNG-deficient patients, who have functional SHM, but impaired CSR, display normal peripheral B cell tolerance. Such findings suggest that altered SHM, not CSR, results in increased autoreactive mature B cell frequency as well as decreased regulatory T cell (Treg) frequency in the periphery, with abnormal Treg phenotype and impaired Treg functions (159). Defective removal of autoreactive mature naïve B cells in *FOXP3* deficient, *DOCK-8* deficient individuals and Wiskott-Aldrich patients revealed that functional Tregs are important for the regulation of peripheral B cell tolerance (160–162). One may think that defective peripheral B cell tolerance in AID-mutated patients is due to the impaired elimination of autoreactive B cells in the bone marrow, since AID deficient patients and *AICDA*-KO mice have defective central B cell tolerance. However, autoreactive B cells migrated from bone marrow can be prevented from colonizing the mature naïve B cell compartment, if the Tregs are functional (102). In addition, patients with autosomal dominant *AICDA* mutations only show defective peripheral B cell tolerance checkpoint, excluding a possible contribution from the bone marrow.

Due to impaired SHM and affinity maturation in AID-deficient patients, antigen expression on follicular dendritic cells in germinal centers is prolonged, leading to sustained B and T cell activation, increased Tfh cell (CD3^+^CD4^+^CXCR5^+^PD-1^+^) production and IL-4, IL-10, IL-21 secretions (163). Increased secretions of IL-4 and IL-21 can disturb the functions of Tregs (164). Increased frequency of circulating Tfh cells induces antibody secretion (165) and may contribute to an altered B cell tolerance as well as the production of IgM serum autoantibodies. Hence, decreased SHM leads to enhanced germinal center reactions, increased Tfh in the periphery, increased cytokine secretion affecting T cell polarization and Treg function. As a result, regulation of peripheral B cell tolerance may in turn be impaired.

AID is physiologically upregulated by NF-κB, STAT6 and Smad transcription pathways and the Th2/Treg cytokines, IL-4, IL-13 and TGF-β, which are important in terms of connecting chronic inflammation to carcinogenesis (68, 166, 167). Hepatocellular carcinoma may be initiated by hepatitis C virus infection. Chronic gastric inflammation and gastric cancer are associated with Helicobacter pylori infection. Chronic inflammation in the colonic epithelium is an important risk factor for colorectal cancer, whereas chronic inflammation in the biliary epithelium is correlated with cholangiocarcinoma. Acid reflux-mediated AID expression contributes to the development of Barret’s adenocarcinoma. In all of those conditions, inflammatory stimulations lead to aberrant AID expression in the epithelium and enhanced susceptibility to mutagenesis (168–171).

Due to Th2/Treg-mediated immunity, AID is upregulated in IgG4-related sialadenitis. AID-positive lymphoid cells, plasma cells and plasmacytoid cells in extra-germinal centers, which indicate broader aberrant AID expression, may be responsible for oncogenesis in patients with IgG4-RD (172). Increased AID activation and DNA damage may also associate ovulation-induced inflammation to carcinogenesis of the fallopian tube epithelium (173). Given these findings, one may think that alternative therapies inhibiting AID function may prevent carcinogenesis in chronic inflammation; however, it should also be kept in mind that it also represents an important defense mechanism at the earlier stages of inflammation.

## Concluding remarks

**Figure 4:**
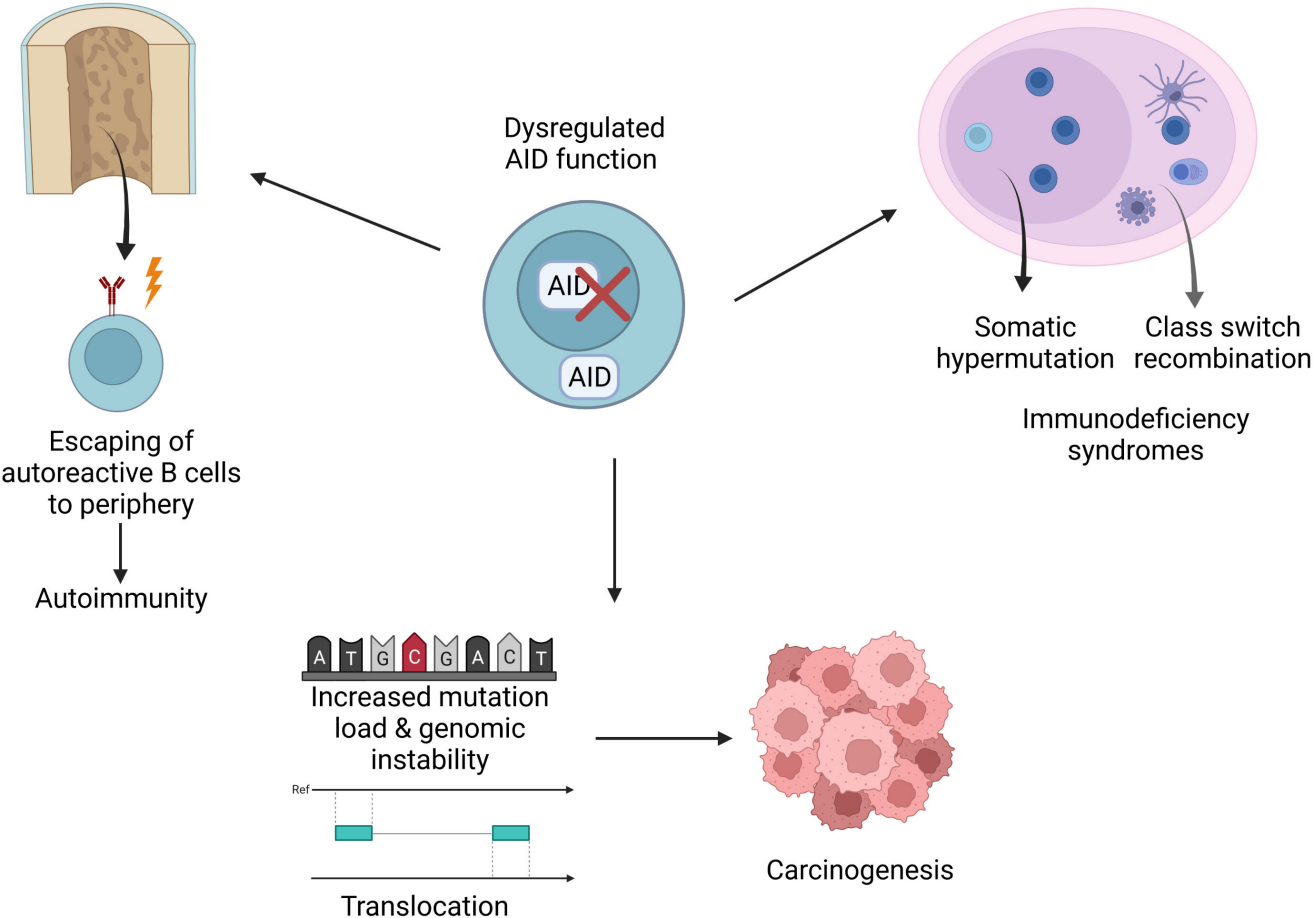
Implications of AID dysregulation. Dysregulations may cause various pathological conditions such as increased mutation load, translocations, disturbed genomic integrity, carcinogenesis, immunodeficiency or autoimmunity.

AID has been known as a key molecule for CSR and SHM in the germinal center for more than two decades. However, recent studies revealed new roles for AID; thus, it can be regarded as a *double-edged* sword given its contributions to epigenetic regulation; achieving a more variable genetic code, which contributes to evolution; establishment of central B cell tolerance; effective humoral responses as well as its role in increased mutation load and carcinogenesis (**Figure 4**). In light of these findings, it is vital to tightly regulate the expression and function of AID. Future studies are needed to better explain the mechanisms underlying the regulation of AID. Moreover, such approaches will pave the way for deciphering the dysregulations that lead to various pathological outcomes such as increased intraclonal heterogeneity, autoimmune diseases, immune deficiency syndromes, lymphoma and other cancers. As a result, these studies have the potential to lead to novel treatment options and therapeutic strategies.

## Author contributions

EÇ and GG prepared the manuscript. Both authors contributed to the article and approved the submitted version.

## Acknowledgments

**Figures 2**–**4** were created with BioRender.com.

## Conflict of interest

The authors declare that the research was conducted in the absence of any commercial or financial relationships that could be construed as a potential conflict of interest.

## Publisher’s note

All claims expressed in this article are solely those of the authors and do not necessarily represent those of their affiliated organizations, or those of the publisher, the editors and the reviewers. Any product that may be evaluated in this article, or claim that may be made by its manufacturer, is not guaranteed or endorsed by the publisher.

## Abbreviations

ABC, Activated B cell like; APOBEC, Apolipoprotein B mRNA editing enzyme, catalytic polypeptide-like; AID, Activation induced cytidine deaminase; BCR, B cell receptor; BER, Base excision repair; CSR, Class switch recombination; CTNNBL1, B-catenin like protein 1; CVID, Common variable immune deficiency; DHL, Double-hit lymphoma; DLBCL, Diffuse large B cell lymphoma; DSB, Double stranded break; GCB, Germinal center B cell like; SHM, Somatic hypermutation; HIGM2, Hyper-IgM syndrome type 2; HNSSC, Head and neck squamous cell cancer; Ig, Immunoglobulin; LPS, Lipopolysaccharide; MMR, Mismatch repair; NHEJ, Nonhomologous end joining; Pax5, Paired box protein 5; PGC, Primordial germ cell; RF, Rheumatoid factor; RSS, Recombination signal sequence; SLE, Systemic lupus erythematosus; SSB, Single stranded break; SSc, Systemic sclerosis; TLR, Toll-like receptor; Treg, Regulatory T cell; UNG, Uracil-DNA glycosylase; UTR, Untranslated region.
